# Impact of anticoagulation and antiplatelet drugs on surgery rates and mortality in trauma patients

**DOI:** 10.1038/s41598-021-94675-7

**Published:** 2021-07-26

**Authors:** Felix M. Bläsius, Markus Laubach, Hagen Andruszkow, Cavan Lübke, Philipp Lichte, Rolf Lefering, Frank Hildebrand, Klemens Horst

**Affiliations:** 1 Department of Trauma and Reconstructive Surgery, University Hospital RWTH Aachen, Pauwelsstraße 30, 52074 Aachen, Germany; 2 Department of Surgical Intensive Care, University Hospital RWTH Aachen, Pauwelsstraße 30, 52074 Aachen, Germany; 3 IFOM—Institute for Research in Operative Medicine, Faculty of Health, Witten/Herdecke University, Ostmerheimer Straße 200, 51109 Cologne, Germany

**Keywords:** Medical research, Outcomes research, Health care, Fracture repair, Geriatrics, Prognosis

## Abstract

Preinjury anticoagulation therapy (AT) is associated with a higher risk for major bleeding. We aimed to evaluated the influence of preinjury anticoagulant medication on the clinical course after moderate and severe trauma. Patients in the TraumaRegister DGU ≥ 55 years who received AT were matched with patients not receiving AT. Pairs were grouped according to the drug used: Antiplatelet drugs (APD), vitamin K antagonists (VKA) and direct oral anticoagulants (DOAC). The primary end points were early (< 24 h) and total in-hospital mortality. Secondary endpoints included emergency surgical procedure rates and surgery rates. The APD group matched 1759 pairs, the VKA group 677 pairs, and the DOAC group 437 pairs. Surgery rates were statistically significant higher in the AT groups compared to controls (APD group: 51.8% vs. 47.8%, p = 0.015; VKA group: 52.4% vs. 44.8%, p = 0.005; DOAC group: 52.6% vs. 41.0%, p = 0.001). Patients on VKA had higher total in-hospital mortality (23.9% vs. 19.5%, p = 0.026), whereas APD patients showed a significantly higher early mortality compared to controls (5.3% vs. 3.5%, p = 0.011). Standard operating procedures should be developed to avoid lethal under-triage. Further studies should focus on detailed information about complications, secondary surgical procedures and preventable risk factors in relation to mortality.

## Introduction

It is well known that older trauma patients have poorer outcomes than younger ones^1^. Physiological changes, comorbidities, nutritional deficits and pre-medication are discussed as reasons for this increased mortality^2–4^. About 4% of trauma patients are on anticoagulation therapy (AT)^5,6^, mainly due to atrial fibrillation and other cardiovascular diseases (e.g. thromboembolic events, prosthetic heart valves)^7–9^. Demographic changes will lead to a further increase in patients on AT^10,11^.

As AT has the potential to increase the risk of major bleeding, it represents a major concern for the post-traumatic course, especially in patients with severe haemorrhage. Several studies have reported preinjury medication with warfarin as an independent predictor of mortality^2,12^. Moreover, impaired physiological reserve due to reduced cardiac function, loss of vessel elasticity and vasoconstriction^3,13^ might mask accelerated blood loss. In up to 20% of geriatric trauma patients, occult hypoperfusion has been described^14^. These risk factors may lead to under-triage, which is associated with a four-fold higher mortality in the geriatric trauma population compared to accurately triaged trauma patients^15^.

Despite the high relevance of AT treatment for the post-traumatic course, the specific impact of different drug groups remains largely unknown. We evaluated the impact of preinjury AT treatment on surgery, thromboembolic events and mortality.

## Materials and methods

### TraumaRegister DGU

The TraumaRegister DGU of the German Trauma Society (Deutsche Gesellschaft für Unfallchirurgie, DGU) was founded in 1993 with the aim of creating a multi-centre database for pseudonymized and standardized documentation of severely injured patients for the purposes of quality assurance and research^16^. Participating hospitals are primarily located in Germany (90%), but a rising number of hospitals from other countries have begun to contribute data as well, to include Austria, Belgium, China, Finland, Luxembourg, Slovenia, Switzerland, The Netherlands, and the United Arab Emirates. Currently, approximately 33,000 cases from more than 650 hospitals are entered into the database per year. Participation in the TR-DGU is voluntary; however, hospitals associated with the TraumaNetzwerk DGU are obligated to enter at least one basic dataset for quality assurance purposes.

Data were collected prospectively over four consecutive time phases from site of injury until discharge from hospital, as follows: (A) prehospital phase, (B) emergency room and initial surgery, (C) intensive care unit (ICU), and (D) discharge. Documentation included detailed information on demographics, injury pattern, comorbidities, pre- and in-hospital management, course of care while in the ICU, relevant laboratory findings (including data on transfusion), and clinical outcome for each individual. Inclusion criteria were: admission to hospital via emergency room with subsequent ICU/intermediate unit care, or entrance to the hospital with vital signs and death prior to admission to the ICU.

Infrastructure for documentation and data management was provided by the Academy for Trauma Surgery (AUC—Akademie der Unfallchirurgie GmbH), a company affiliated with the German Trauma Society. Scientific leadership draws from the Committee on Emergency Medicine, Intensive Care and Trauma Management (Sektion NIS) of the German Trauma Society. Participating hospitals submitted pseudonymized data into a central database via web-based application. Scientific data analysis was approved according to a peer review procedure established by the NIS Committee .

The present study is in line with the publication guidelines of the TR-DGU and registered as TR-DGU project ID 2018-006.

### Definitions

The patients were treated according to the German Level 3 guideline on the treatment of patients with severe/multiple injuries (Version 1 from 2011 and 2016 Update)^17^. Injuries were coded according to the Abbreviated Injury Scale (AIS, Version 2005/2008, Association for the Advancement of Automotive Medicine, Barrington, IL). The severity of injuries was recorded according to the AIS as 1 (minor), 2 (moderate), 3 (severe, not life threatening), 4 (serious, life-threatening), 5 (critical, survival uncertain) and 6 (maximum, currently untreatable). Overall injury severity was calculated by the Injury Severity Score (ISS) as described by Baker et al.^18^. The outcome after severe brain damage has been assessed by using the Glasgow Outcome Scale (GOS)^19^.

Hypotension was defined as systolic blood pressure < 90 mmHg at hospital admission. Thrombembolic events included deep vein thrombosis, pulmonary embolism and ischemic stroke during hospital stay. The definition of coagulopathy included at least one of the following criteria: (A) International normalized ratio (INR) > 1.4, (B) Activated partial thromboplastin time (aPTT) ≥ 40 s, and (C) Quick ≤ 60%^20^, at hospital admission. Acidosis was defined as a pH value < 7.35 in the first blood gas analysis within the resuscitation room. A mass transfusion was coded, if ≥ 10 units of packed red blood cells (PRBC) were transfused within 24 h.

Mortality was reported as early (< 24 h) and total in-hospital mortality. Furthermore, we used the Revised Injury Severity Classification score, version II (RISC II) to predict the risk of death in severely injured patients that were primarily admitted to one of the reporting trauma centres^21^.

The variable “surgery” included all surgical procedures performed within the hospital stay. Emergency Surgery procedures (ESP) included:

- Decompressive craniectomy
- Laminectomy
- Emergency thoracotomy
- Laparotomy
- Revascularisation
- Embolisation
- Stabilisation of pelvic fractures
- Stabilisation of extremity fractures

Anticoagulants and antiplatelet drugs investigated in this study are presented in Table 1.

**Table 1:** The classes of anticoagulants and antiplatelet agents and the agents included.

Antiplatelet drugs (APD)	Acetylsalicylic acid P2Y_12_ receptor blockers (Clopidogrel, Prasugrel, Ticagrelor) Glycoprotein IIb/IIIa inhibitors (Abciximab, Eptifibatide, Tirofiban)
Vitamin K antagonsits (VKA)	Phenprocoumon Warfarin
Direct oral anticoagulants (DOAC)	Factor IIa inhibitor (Dabigatran) Factor Xa inhibitors (Apicaban, Edoxaban, Otamixaban, Rivaroxaban)

### Inclusion criteria

The following inclusion criteria were applied:

- AIS ≥ 3 in at least one body region
- Age ≥ 55 years
- Treated in a German hospital
- Standard data entry form (Version 2015, admissions from 2015 to 2018)
- Data availability regarding AT
- Early (< 48 h) transfer out excluded

**Figure 1:**
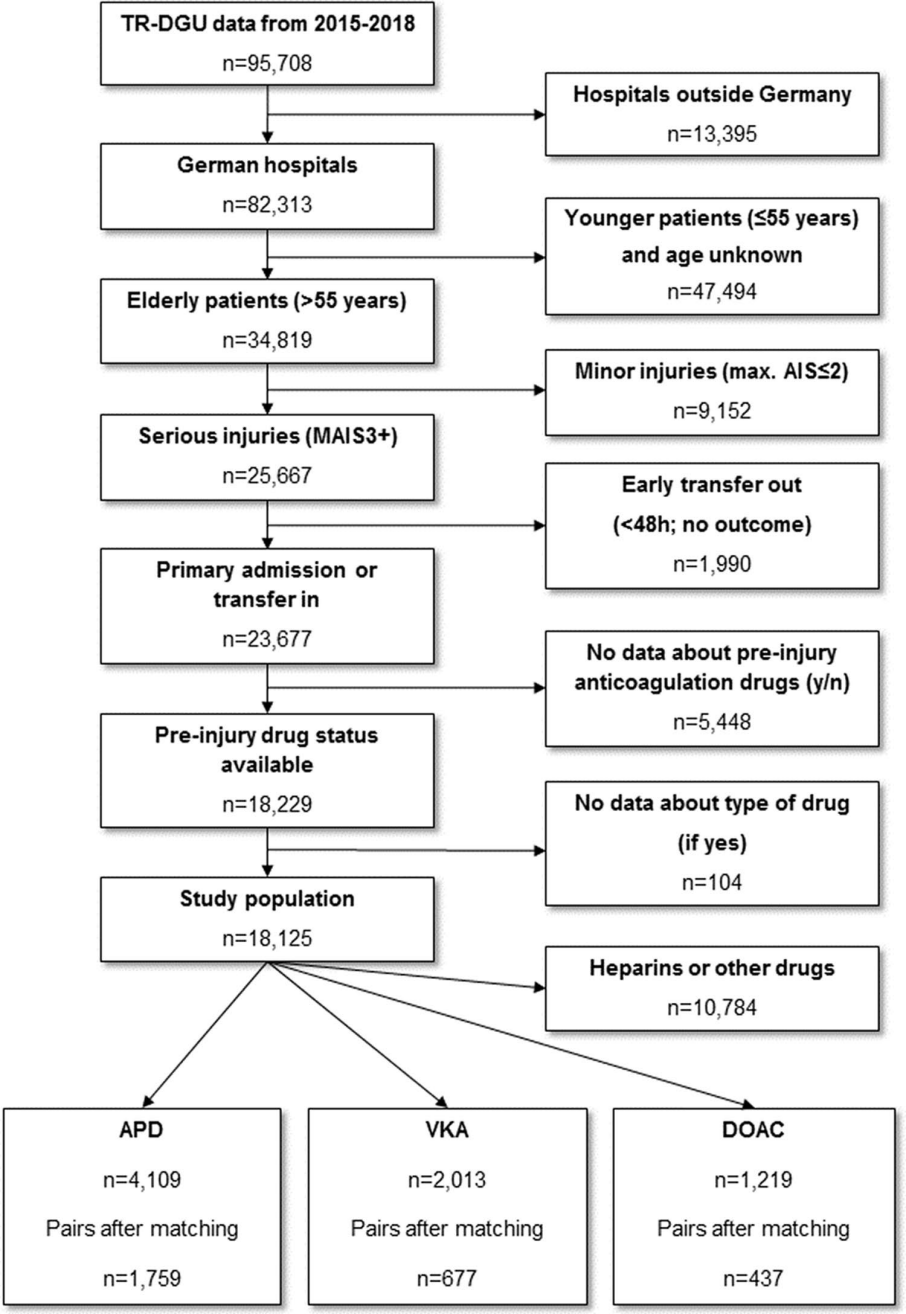
Study flow chart illustrating the selection of patients.

Patients were then grouped according to their AT-medication (APD, VKA or DOAC). Patients who took a combination of two drugs were excluded. Each group of patients was matched to control patients who did not receive an anticoagulation drug (Fig. 1).

### Matched-pair analysis

Patients with and without AT were matched according to the following nine criteria (exact match):

- Age groups (years): 55–59, 60–64, 65–69, 70–74, 75–79, 80–84, 85–89, ≥ 90
- Sex (male vs. female)
- AIS_Head_ (0–1/2/3/4/5–6)
- AIS_Thorax_ (0–1/2/3/4/5–6)
- AIS_Abdomen_ (0–1/2/3/4/5–6)
- AIS_Extremities_ (0–1/2/3/4/5–6)
- Pre-trauma ASA classification (1–2 vs. 3–4)
- Type of admission (primary vs. secondary)
- Systolic blood pressure on hospital admission (divided into four categories (mmHg): < 90, 90–110, 111–179, ≥ 180)

### Statistics

The subgroups resulting from the matching protocol according to the AT taken were compared and analyzed individually to controls. No statistical intergroup comparisons were performed. Categorical variables are presented as percentages if the underlying total is obvious. Metric data are presented as mean and median with standard deviation. Differences in categorical and metric variables were evaluated with the McNemar test and Wilcoxon test for paired data, respectively. A two-sided p-value < 0.05 was considered significant. The interpretation of results should generally focus on the clinical relevance of an observed difference rather than p-values alone. All statistical analyses were performed using SPSS statistical software (SPSS 25; IBM Inc., Armonk, NY, USA).

### Consent for publication

The TR-DGU gave permission for publication. All authors read and approved the final manuscript and gave permission for publication.

## Results

A total of 18,125 patients met the inclusion criteria. The matched-pair analyses were performed with 5,746 patients (2,873 pairs), resulting in 1,759, 677 and 437 pairs with APD, VKA and DOAC pre-medication, respectively. By definition, the pairs were identical in terms of the nine matching factors. Table 2 summarises each matched-pair group’s baseline characteristics and injury mechanisms.

**Table 2:** Demographics, trauma mechanisms, injury pattern and outcome. Continuous values are presented as mean/median (SD).

	APD group 1,759 pairs			VKA group 677 pairs			DOAC group 437 pairs		
	APD	Control	p-value	VKA	Control	p-value	DOAC	Control	p-values
**Demographics**									
Male (%)	64.2			60.7			56.3		
Age (mean/median) (SD)	75.1/76 (9.6)	75.0/76 (9.7)		76.7/78 (8.5)	76.7/77 (8.6)		77.1/78 (9.1)	77.0/78 (9.2)	
Age > 65 years (%)	82.3			89.1			88.6		
**Injury mechanism**									
Blunt trauma (%)	98.3	98.7	p = 0.33	98.5	97.9	p = 0.54	98.8	97.2	p = 0.14
Low fall < 3 m (%)	54.6	50.8	p = 0.025	65.8	57.2	p = 0.001	62.0	56.6	p = 0.112
High fall > 3 m (%)	10.5	12.1	p = 0.15	7.5	10.6	p = 0.056	8.5	11.1	p = 0.21
Car (%)	10.4	12.0	p = 0.13	11.2	8.5	p = 0.10	13.6	10.2	p = 0.14
Motorcycle (%)	6.0	4.2	p = 0.017	3.1	3.7	p = 0.65	2.8	3.0	p = 0.84
Bicycle (%)	9.3	9.9	p = 0.57	6.1	11.2	p = 0.001	7.4	7.7	p = 0.90
Pedestrian (%)	5.0	5.4	p = 0.59	3.0	2.8	p = 0.87	1.6	7.0	p < 0.001
**Basic parameter**									
Primary admission (%)	92.9			90.7			92.0		
LOS, mean/median (SD), d	15.3/12 (13.6)	15.3/12 (14.1)	p = 0.87	15.4/12 (13.5)	14.9/12 (12.6)	p = 0.97	14.3/11 (11.7)	14.5/12 (11.8)	p = 0.96
Duration on ICU, mean/median (SD), d	5.5/2 (8.5)	5.5/2 (8.4)	p = 0.87	6.4/3 (9.3)	6.1/3 (8.9)	p = 0.46	5.7/2 (9.2)	5.6/2 (8.6)	p = 0.74
Intubation, mean/median (SD), d	2.1/0 (6.2)	2.3/0 (6.1)	p = 0.39	2.9/0 (7.3)	2.5/0 (5.7)	p = 0.44	2.2/0 (5.9)	2.5/0 (6.6)	p = 0.083
ASA Score 3–4 (%)	34.8			52.6			56.8		
ISS, mean/median (SD)	17.2/16 (7.2)	17.3/16 (7.8)	p = 0.93	18.4/17 (8.3)	18.9/17 (9.1)	p = 0.38	17.5/16 (8.1)	17.6/16 (8.8)	p = 0.63
AIS_Extremity_ ≥ 3 (%)	20.2			17.1			19.5		
AIS_Abdomen_ ≥ 3 (%)	2.4			1.6			1.1		
AIS_Thorax_ ≥ 3 (%)	37.6			32.8			35.0		
AIS_Head_ ≥ 3 (%)	50.7			57.8			53.5		
Total in-hopistal mortality (%)	14.0	13.6	p = 0.73	23.9	19.5	p = 0.026	19.0	19.7	p = 0.84
Early in-hospital mortality, < 24 h (%)	5.3	3.5	p = 0.011	8.7	7.5	p = 0.49	5.7	6.9	p = 0.58
RISC II prognosis (%), primary admissions only	13.5	13.1	p = 0.83	25.6	19.4	p < 0.001	19.4	19.5	p = 0.17
Level I trauma center (%)	52.5	49.4		56.6	57.0		48.5	56.1	

Length of stay (LOS), ICU length of stay and intubation rates showed no differences within the three matched-pair analyses (Table 2). Patients on APD treatment had a significantly higher early in-hospital mortality rate (< 24 h) compared to the controls (5.3% vs. 3.5%, p = 0.011). However, total in-hospital mortality did not differ (14.0% vs. 13.6%, p = 0.73) (Table 2). The APD group had higher surgery rates compared to the controls (51.8% vs. 47.8%, p = 0.015), whereas ESP rates and time until ESP showed no differences. Prehospital hypotension incidence, coagulopathy, the number of received PRBC and prothrombin complex concentrate (PCC) transfusions were comparable between groups (Table 3).

**Table 3:** Clinical data of APD group. Continuous values are presented as mean/median (SD).

	Antiplatelet drug		Data available (n)	*p*-value
	Yes	Control		
HEMS (%)	13.2	12.8	3,157	0.74
CT (%)	87.5	86.0	3,518	0.20
WBCT (%)	65.9	66.7	3,500	0.61
Emergency surgical procedures (%)	17.8	18.7	3,398	0.47
Time to emergency surgical procedures, mean/median (SD), min	75.9/77 (25.4)	70.0/72.5 (27.4)	375	0.058
Surgery (%)	51.8	47.8	3,518	0.015
Packed red blood cells (%)	3.6	2.8	3,515	0.21
Massive transfusion (%)	0.2	0.1	3,515	0.69
Fresh frozen plasma (%)	1.3	1.1	3,515	0.76
Prothombin complex concentrates (%)	1.9	1.2	1,421	0.27
Coagulopathy (%)	6.0	5.2	3,398	0.28
…INR, mean/median (SD)	1.09/1.02 (0.42)	1.07/1.02 (0.18)	3,397	0.59
Platelet count in ER (per µl)	216,246/207,500 (90,176)	211,640/204,000 (73,646)	1,440	0.41
Hypotension – pre-hospital (%)	4.8	3.8	2,922	0.16
Hypotension in ER (%)	2.4	2.0	3,367	0.42
Tranexamic acid (%)	3.3	2.3	3,068	0.11
Prehospital fluid resuscitation, mean/median (SD), ml	529/500 (398)	586/500 (438)	2,994	0.001
ER fluid resusctitation mean/median (SD), ml	760/500 (875)	837 /500 (1172)	1,253	0.27
Acidosis (%)	7.7	7.5	2,713	0,88
Platelet count in ER (per µl)	216,246/207,500 (90,176)	211,640/204,000 (73,646)	1,440	0.41
Thrombembolic event (%)	3.6	3.2	1,443	0,73
Glasgow outcome scale 4–5 (%)	77.0	77.0	3,509	0.99

Total in-hospital mortality (23.9% vs. 19.5%, p = 0.026), RISC II prognosis (25.6% vs. 19.4%, p < 0.001) (Table 2) and INR values (2.36 ± 1.07 vs. 1.08 ± 0.2, p < 0.001) were higher in the VKA group. Furthermore, this group presented with coagulopathy (84.7% vs. 6.1%, p < 0.001) and received PCC more frequently (26.3% vs. 1.9%, p < 0.001), and surgical treatment was more frequent compared to the controls (52.4% vs. 44.8%, p = 0.005). However, no differences in ESP rates were found. Patients on VKA less often presented with prehospital hypotension (2.9% vs. 5.5%, p = 0.038) (Table 4).

**Table 4:** Clinical data of VKA group. Continuous values are presented as mean/median (SD).

	Vitamin K antagonist		Data available (n)	*p*-value
	Yes	Control		
HEMS (%)	13.4	14.3	1,183	0.64
CT (%)	86.0	85.7	1,354	0.88
WBCT (%)	60.2	61.4	1,344	0.66
Emergency surgical procedures (%)	17.9	18.2	1,309	0.50
Time to emergency surgical procedures, mean/median (SD), min	68.4/69.5 (27.7)	71.6 /73 (24.5)	159	0.44
Surgery (%)	52.4	44.8	1,354	0.005
Packed red blood cells (%)	3.8	2.2	1,352	0.083
Massive transfusion (%)	0.1	0.1	1,352	
Fresh frozen plasma(%)	1.6	0.6	1,352	0.070
Prothombin complex concentrates (%)	26.3	1.9	612	< 0.001
Platelet count in ER (per µl)	201,023/195, 500 (63,724)	211,285/206,000 ()81,792	626	0.044
Coagulopathy (%)	84.7	6.1	1,305	< 0.001
…INR, mean/median (SD)	2.36/2.22 (1.07)	1.08/1.03 (0.20)	1,303	< 0.001
Acidosis (%)	10.1	7.0	1,064	0.071
Hypotension – prehospital (%)	2.9	5.5	1,093	0.038
Hypotension in ER (%)	2.7	2.8	1,279	0.88
Tranexamic acid (%)	2.5	2.6	1,143	0.91
Prehospital fluid resuscitation, mean/median (SD), ml	558/500 (424)	576/500 (482)	1,124	0.75
ER fluid resuscitation mean/median (SD), ml	720/500 (714)	728/500 (921)	537	0.21
Thrombembolic event (%)	5.0	3.2	607	0.27
ER fluid mean/median (SD), ml	720/500 (714)	728/500 (921)	537	0.21
Glasgow outcome scale 4–5 (%)	67.7	67.8	1,347	0.97

DOAC therapy was associated with a significant increase in the coagulopathy rate (37.5% vs. 5.2%, p < 0.001), number of received PCC transfusions (10.2% vs. 0.5%, p < 0.001) and incidence of surgical procedures (52.6% vs. 41.0%, p = 0.001). INR values were significantly higher in the DOAC group compared to the controls (1.41 ± 0.63 vs. 1.09 ± 0.39, p < 0.001). However, no differences in ESP rate, incidence of prehospital hypotension or thromboembolic events and PRBC transfusion rates were observed (Table 5). Furthermore, no differences could be seen in total in-hospital mortality, early mortality (< 24 h) and RISC II prognosis (Table 2).

**Table 5:** Clinical data of DOAC group. Continuous values are presented as mean/median (SD).

	DOAC		Data available (n)	*p*-value
	Yes	Control		
HEMS (%)	13.0	12.8	775	0.92
CT (%)	88.3	85.4	874	0.19
WBCT (%)	62.2	65.7	866	0.28
Emergency surgical procedures (%)	14.4	14.9	848	0.85
Time to emergency surgical procedures, mean/median (SD), min	77.7/83.5 (26.4)	77.8/75 (23.3)	83	0.82
Surgery (%)	52.6	41.0	874	0.001
Packed red blood cells (%)	3.4	1.8	874	0.14
Massive transfusion (%)	0	0.2	437	
Fresh frozen plasma (%)	1.1	0.7	874	0.48
PProthombin complex concentratesC (%)	10.2	0.5	376	< 0.001
Platelet count in ER (per µl)	206,952/203,000 (69,450)	218,785/202,000 (99,165)	386	0.42
Coagulopathy (%)	37.5	5.2	842	< 0.001
…INR, mean/median (SD)	1.41/1.20 (0.63)	1.09/1.03 (0.39)	842	< 0.001
Acidosis (%)	9.7	8.7	643	0.63
Hypotension – prehospital (%)	4.0	6.9	711	0.084
Hypotension in ER (%)	1.9	2.3	837	0.49
Tranexamic acid (%)	3.8	2.6	749	0.37
Prehospital fluid, mean/median (SD), ml	501/500 (416)	546/500 (427)	726	0.11
ER fluid mean/median (SD), ml	712/500 (667)	740/500 (636)	342	0.28
Thrombembolic event (%)	2.7	2.1	382	0.70
Glasgow outcome scale 4–5 (%)	69.8	69.7	872	0.96

## Discussion

Demographic changes and comorbidities will result in an increased incidence of geriatric trauma patients with AT. The recent approval of novel anticoagulants has further increased the diversity of AT medication. Trauma care of these patients is still debated, with inconsistent data regarding the clinical course after moderate or severe trauma. To elucidate to what extent different anticoagulants (APD, DOAC or VKA) affect the clinical course after severe trauma, we performed a retrospective study based on trauma registry data.

The main findings of our matched-pair analyses can be summarised as follows:

1. AT did not influence the incidence of ESP but resulted in higher surgery rates over the further clinical course in all medication groups.
2. In contrast to APD and VKA, DOAC affected neither early nor total in-hospital mortality.
3. Coagulopathy rates and PCC transfusion rates were significantly higher in patients with both DOAC and VKA treatment.

Patients with VKA and DOAC treatment had significantly higher coagulopathy rates. As increased INR values represent a VKA treatment goal and have also been associated with DOAC treatment^22^, this supported our expectations. Surprisingly, about 15% of VKA patients had an INR < 1.5 and were thus in the non-therapeutic range. This observation could be explained by two main reasons. First, a non-adherence to VKA therapy is described in up to 20% of the respective patients^23^. On the other hand, monitoring in the patient group we investigated (patients > 75 years of age) is mostly performed by the primary care physician. Monitoring takes place once a month. Between these monitoring appointments, non-therapeutic and overshooting INR values may occur. Moreover, only a few patients of this age perform close-meshed self-monitoring (e.g., CoaguChek or INRatio2 point-of-care-systems at least once a week)^24^.

As coagulopathy is part of the “lethal triad” and typically associated with severe bleeding, we assessed preclinical and in-hospital hypotension rates. Interestingly, the controls showed a significantly higher prehospital hypotension rate compared to patients with VKA treatment. This might be explained by the higher risk of vascular calcification in patients with low vitamin K levels^25^. Vitamin K is needed to synthesise matrix glad protein (MGP), a natural calcification inhibitor. In mice, targeted deletion of the MGP gene resulted in rapid and complete arterial calcification, resulting in death by 6 weeks^26^. Vitamin K antagonism with warfarin, which antagonises the vitamin K-dependent MGP carboxylation, leads to rapid arterial calcification in rats^27^. Furthermore, diets high in vitamin K have been shown to reverse aortic calcification and improve arterial elasticity in warfarin-treated rats, suggesting that the calcification in response to warfarin treatment is due to the inhibition of the vitamin K-dependent MGP γ-carboxylation^28^. Due to vessel calcification, elasticity decreases and hypertension might develop. The higher hypertension risk in patients with low vitamin K and D levels has been described earlier^29^. Thus, the observed difference might be biased by the patients’ comorbidities. Pathological hypertension may affect patients on VKA treatment. In cases of blood loss, these patients might present with physiological blood pressure rather than hypotension. Although vitamin K’s role in vascular calcification has been demonstrated in animal and in vitro studies, evidence from human studies needs to be further elucidated. Our data may present useful references for the trauma population.

As haemostasis control is the goal in anticoagulated patients during resuscitation, we also investigated PCC, FFP and PRBC transfusion rates. PCC transfusion rates were significantly increased in patients with VKA and also with DOAC treatment. This was expected as guidelines recommend PCC application for reversal of VKA- and DOAC-associated coagulopathy in trauma patients^17^. As ESP rates and time to ESP did not differ between patients on AT and controls, the decision to perform ESP likely was not influenced by AT and the transfusion therapy was sufficient to achieve haemostasis in most patients with VKA and DOAC treatment. This supports treatment algorithms, which recommend antagonisation of known pharmacological coagulopathies before surgery^30^.

We found a significantly higher incidence of overall surgical procedures in patients with AT in all groups. As the AIS was comparable between AT and control patients, different injury severities as a reason can be excluded. We assume that AT-related development of clinically relevant haematomas, ongoing bleeding or compartment syndromes were indications for the higher number of operations. This has been reported to cause surgical interventions and is likely to contribute to higher surgical rates in AT patients^31,32^. Furthermore, several studies have associated relevant anticoagulation with prolonged wound drainage, which is conducive for infection development, due to hematoma formation, which predisposes towards infection^33,34^. More numerous persistent drainage or infections, although mostly superficial, may have contributed to the observed higher surgical intervention rates in all three treatment groups compared to controls^34^. However, due to the TraumaRegister DGU design, we were unable to verify an increase in operations due to one of the above-mentioned reasons.

Although surgical procedures are indications for AT reversal^35^, PCC application may cause severe side effects. PCC application and a delayed AT restart after surgery were thought responsible for higher incidences of thromboembolic events in the 1990s and early 2000s^36^. Our results did not show higher thromboembolic event rates in trauma patients on AT, confirming Majeed et al.’s findings on thromboembolic events after PCC application^37^. A possible explanation might be that modern PCCs have a higher degree of security, which has been attributed to the inclusion of coagulation inhibitors such as protein Z and heparin. As a result, a balance of pro- and anti-coagulatory effects seems to have been achieved^38^.

Despite the different overall surgical rates, LOS and ICU treatment duration did not differ between the AT patients and the controls. Protocolised daily examinations during the ICU stay might have led to early identification of patients at risk for further interventions and surgery^39,40^. Improved general status might have resulted in transfer to the normal ward, so additional surgical interventions did not prolong the ICU stay. Moreover, the severity of these surgical interventions might not have been as invasive as those needed to stabilise the patient, so renewed ICU treatment was unnecessary. As the patients on VKA and APD showed a higher total in-hospital mortality rate, it might be assumed that the increased number of deceased patients resulted in a LOS bias in these patients. Nevertheless, due to the low mortality rates, especially in APD patients, this potential bias seems negligible.

For mortality analysis, we distinguished between early and total in-hospital mortality as previously recommended^41^. Early in-hospital mortality was significantly higher in patients on APD compared to the controls (5.3% vs. 3.5%, p = 0.011). This difference diminished during the hospital stay and culminated in nonsignificant differences in the total in-hospital mortality rate, possibly due to the beneficial effects of APD on the immune system during the further clinical course of critically ill patients. A lower rate of post-traumatic acute respiratory distress syndrome, sepsis and multiple organ failure has been published^42,43^. Thus, some protective characteristics of ADP might have contributed to lower overall mortality during the later clinical course (> 24 h). The diagnosis of haemorrhagic shock, characterised by hypotension, does not seem to be causal for early in-hospital mortality in APD patients. Head trauma is common in multiply injured patients and was a leading injury (more than 50%) in each medication group. A meta-analysis by Batchelor et al. reported a trend towards increased mortality rates in head trauma patients on APD medication, due to intracranial haematoma enlargement^44,45^. In contrast, DOAC-associated intracranial haemorrhage resulted in smaller baseline haematoma volumes and a lower likelihood of haematoma expansion compared to VKA-associated haemorrhage^46^. Hence, the in-hospital mortality rate was lower in DOAC-associated intracranial haemorrhage (adjusted risk difference in favour of DOACs, 5.7%). This difference was accentuated among patients pre-treated with antiplatelet therapy in addition to VKA treatment (adjusted risk difference in favour of DOACs, 15%)^47^. Beneficial effects of DOAC have also been described by Mullins et al., who found no difference in 30-day mortality in a matched-pair analysis of 124 patients with proximal femoral fractures^48^. Although no statistical comparison of mortality rates between analyses was permitted because of the isolated matched-pair analyses, cautious comparisons can be made because the matched-pairs had markedly comparable baseline data. Our findings support recent safety studies reporting bleeding complications and thromboembolic events in patients on DOACs compared to VKA treatment^49^. A potential explanation might be the different effects on the coagulation system^50^. Whereas exposed tissue factor induces thrombin generation that can overcome DOACs’ inhibitory effects, the markedly reduced levels of FVIIa by VKA impair this response and increase the likelihood of severe bleeding even in modest trauma. Althoughs statistical comparison of matched-pair analyses was not feasible within this study, the observed findings might support previous findings: PCC rates—as a potential reversal therapy—were markedly increased in VKA compared to controls (Table 4) and were also 2.5-fold higher compared to differences between DOACs and their controls (Table 5). Yet, furher studies need to investigate this findings in more detail. In line with previous studies, patients with VKA treatment showed a significantly higher total mortality compared to controls (23.9% vs. 19.5%, p = 0.026)^51^. Other reasons include higher thromboembolic event rates due to VKA reversal and higher surgery rates in patients with VKA treatment^52^. Although the present study did not reveal higher thrombembolic event rates in patients on AT, higher surgery rates in patients with VKA treatment were observed. It is likely that bleeding complications during the further clinical course potentially influence the total in-hospital mortality in this subgroup, and careful monitoring of patients with VKA treatment within the Tertiary Trauma Survey remains crucial for early identification of patients at risk of ongoing bleeding^53^. Further studies exploring traumatic bleeding in patients with non-vitamin K oral anticoagulants are warranted^54^.

The large number of included patients, the strict inclusion criteria and the rigorous matching protocol were sufficient to provide clinically relevant data for geriatric trauma patients on AT. There were some limitations, however. Other combinations of matching criteria and extended matching criteria would have been possible, but additional criteria would have resulted in a loss of statistical power. The criteria were therefore carefully selected and, in our opinion, represent a good balance between goal-oriented matching results and statistical power. Although geriatric trauma is usually defined as patients older than 65 years^55^, vascular diseases leading to AT by DOAC predominantly occur by the age of 55 years^56^. Thus, this lower age threshold was included. Additionally, patients who died before hospital admission are not included in the TR-DGU. Therefore, preclinical causes of death cannot be investigated. This risks overlooking AT-related prehospital deaths of trauma patients. Another limitation results from the reduced data set (standard data entry form) of the TR-DGU that we used. Detailed information on the diseases for which the medications were prescribed or specific laboratory parameters (e.g. kidney function) were not recorded in this data set. No detailed information about comorbidities leading to AT and their potential influence on the clinical course and mortality was available. Moreover, registry studies are restricted to documented data quality. The TR-DGU is not designed to monitor anticoagulant use, and time since the last medication ingestion was not recorded. Additionally, subgroup analyses for different DOAC (Factor IIa and Xa inhibitors), PCC (three-factor PCC; four-factor PCC and activated PCC), and APD (single vs. dual APD treatment) could not be performed due to missing data. Nevertheless, the TR-DGU data quality is very high.

## Conclusion

Although not influencing ESP or the time span until ESP, AT significantly affects the entire clinical course with higher overall surgical rates compared to controls. Further research should focus on improvement of diagnostic procedures and treatment algorithms for the early phase of trauma care. Prospective studies are needed to investigate possible risk factors and complications in trauma patients with AT to ultimately reduce the higher overall surgical and mortality rates.

## Data Availability

Data are provided by the TraumaRegister DGU. Data are available from the TraumaRegister DGU for researchers who meet the criteria for access to confidential data.

## Abbreviations

AT: Anticoagulant therapy
AIS: Abbreviated injury scale
APD: Antiplatelet drugs
ASA: American Society of Anesthesiologists
AUC: AUC—Academy for Trauma Surgery
DGU: German Trauma Society
DOAC: Direct oral anticoagulant
ESP: Emergency surgical procedures
ICU: Intensive Care Unit
ISS: Injury Severity Score
LOS: Length of stay
PCC: Prothrombin complex concentrates
PRBC: Packed red blood cells
RISC II: Revised Injury Severity Classification Score II
SD: Standard deviation
TR-DGU: TraumaRegister DGU
TTS: Tertiary Trauma Survery
VKA: Vitamin K antagonist
